# Efficacy of ventilator for patients with atelectasis: A systematic review protocol of randomized controlled trials

**DOI:** 10.1097/MD.0000000000017259

**Published:** 2019-09-27

**Authors:** Zhi-Guo Wang, Jian-Rong Sun, Hai-Wang Sha

**Affiliations:** a Department of Elderly Respiratory Medicine, Cardiovascular and Cerebrovascular Specialist Ward Affiliated to Yanan University; b Department of Elderly Respiratory Medicine, Dongguan Hospital of Yanan University Affiliated Hospital; c Surgical Intensive Care Center, Yanan University Affiliated Hospital, Yan’an, China.

**Keywords:** atelectasis, efficacy, safety, ventilator

## Abstract

**Background:**

This study aims to assess the efficacy and safety of ventilator for the treatment of atelectasis.

**Methods:**

We will search Cochrane Library, MEDLINE, EMBASE, CINAHL, EBSCO, Chinese database Chinese Biomedical Literature Database, China National Knowledge Infrastructure, and Wanfang data from inceptions to June 30, 2019 without language limitations. We will include randomized controlled trials (RCTs) of ventilator on evaluating the efficacy and safety of ventilator for atelectasis. We will use Cochrane risk of bias tool to assess the methodological quality for all included RCTs. RevMan 5.3 software will be used for statistical analysis.

**Results:**

The primary outcome is lung function. The secondary outcomes comprise of airway pressure, mean arterial pressure, arterial blood gas, heart rate, respiratory rate, oxygen saturation, and adverse events.

**Conclusion:**

The findings of this study will provide most recent evidence of ventilator for the treatment of atelectasis.

**Systematic review registration:**

PROSPERO CRD42019139329.

## 1 Introduction

Atelectasis is one of the most common respiratory disorders, which often manifests as difficulty breathing, rapid, shallow breathing, wheezing, and cough.^[1–3]^ Most patients with atelectasis appears and results in transient lung dysfunction 24 hours post-surgery.^[4–6]^ It has been estimated that the incidence of atelectasis is 90% in patients receiving general anesthesia.^[7–10]^ This condition consists of obstructive, nonobstructive, postoperative, and rounded atelectasis.^[11]^ Several factors are responsible for this disorder, including compressive atelectasis, resorptive atelectasis, and impaired lung surfactant production or function.^[12–14]^

Several managements are helping to prevent and treat this disorder, including positive pressure, ventilation strategy, dornase alpha, and ventilator, especially for ventilator.^[15–24]^ However, no systematic review was conducted to assess its efficacy and safety of ventilator for the treatment of patients with atelectasis. Therefore, this study will systematically assess the efficacy and safety of ventilator for atelectasis.

## 2 Methods

### 2.1 Objective

This study aims to investigate the efficacy and safety of ventilator for the treatment of atelectasis.

### 2.2 Study registry

This study protocol has been registered on PROSPERO CRD42019139329. It has been reported according to the Cochrane Handbook for Systematic Reviews of Interventions and the preferred reporting items for systematic reviews and meta-analysis protocol (PRISMA-P) statement guidelines.^[25]^

### 2.3 Inclusion criteria for study selection

#### 2.3.1 Types of studies

Randomized controlled trials (RCTs) assessing the efficacy and safety of ventilator for atelectasis without any language restrictions. Any other studies, such as case reports, non-RCTs, and quasi-RCTs will all be excluded.

#### 2.3.2 Types of participants

Patients of any age and gender with atelectasis, in any setting, irrespective primary diagnosis will be considered for inclusion.

#### 2.3.3 Types of interventions

Any types of ventilator treatment as an experimental intervention will be included.

Control intervention can be any kinds of medications or therapies, except the ventilator treatment.

#### 2.3.4 Types of outcome measurements

The primary outcome is lung function, as measured by forced expiratory volume in 1 second, or other tools. The secondary outcomes include airway pressure, mean arterial pressure, arterial blood gas, heart rate, respiratory rate, and oxygen saturation.

### 2.4 Search methods for identification of studies

#### 2.4.1 Electronic searches

We will retrieve from following electronic databases of Cochrane Library, MEDLINE, EMBASE, CINAHL, EBSCO, Chinese database Chinese Biomedical Literature Database, China National Knowledge Infrastructure, and Wanfang databases from inceptions to June 30, 2019 without language limitations. The search strategy for Cochrane Library is presented in Table 1. We will also apply similar strategies to any other electronic databases.

**Table 1:**
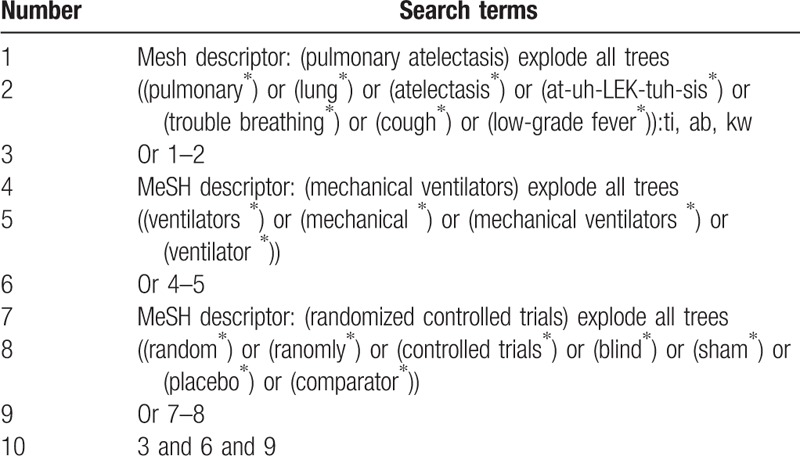
Search strategy for Cochrane Library database.

#### 2.4.2 Search for other resources

We will also retrieve other literature sources, such as clinical registry, reference list of relevant reviewers, and dissertations.

### 2.5 Data collection and analysis

#### 2.5.1 Study selection

Two authors will independently evaluate titles and abstracts of all studies identified. Any divergences between 2 authors will help to solve them by a third author with discussion. After the initial selection, all remaining studies will be read by full-text to identify those are eligible for all inclusion criteria. The whole process of study selection follows and presents in the PRISMA flow chart.

#### 2.5.2 Data collection and management

In this study, 2 authors will carry out data extraction independently. A third author will help to solve disagreements between 2 authors by discussion. Details of all eligible studies will be extracted and summarized using a data extraction form. This form includes title, first author, year of publication, region, disease diagnosis, inclusion and exclusion criteria, patient characteristics, study design, sample size, study methods, treatment details, outcome measurements, and adverse events.

#### 2.5.3 Risk of bias assessment

Two authors will independently evaluate the risk of bias for each eligible study using the Cochrane Handbook for Systematic Reviews of Interventions tool. It has 7 aspects, and each aspect is further categorized into 3 types: low risk of bias, unclear risk of bias, and high risk of bias. Any disagreements will be resolved by a third author via discussion.

#### 2.5.4 Measurement of treatment effect

We will express enumeration data using risk ratio and 95% confidence intervals. We will present continuous data as mean difference or standardized mean difference and 95% confidence intervals.

#### 2.5.5 Dealing with missing data

We will contact corresponding authors of primary studies if there are insufficient or missing data. We will discuss it's impacts of the outcome results if we can not achieve the missing data.

#### 2.5.6 Assessment of heterogeneity

We will use *I*^2^ test for heterogeneity among eligible studies. The value of *I*^2^ ≤ 50% indicates acceptable heterogeneity, and a fixed-effects model will be applied. The value of *I*^2^ > 50% shows significant heterogeneity, and a random-effects model will be applied. At the same time, the subgroup analysis will be performed.

#### 2.5.7 Data synthesis

RevMan 5.3 software is used for statistical analysis. If there is acceptable heterogeneity, we will apply a meta-analysis. If there is significant heterogeneity, we will perform meta-analysis according to the results of subgroup analysis. We will not pool the data and carry out meta-analysis if there is still significant heterogeneity. We will report a narrative summary instead.

#### 2.5.8 Subgroup analysis

We will carry out subgroup analysis in accordance with the different treatments, controls and outcome measurements.

#### 2.5.9 Sensitivity analysis

We will perform sensitivity analysis to check the robustness of pooled results according to the methodological qualities, and statistical models.

#### 2.5.10 Publication biases

We will carry out the Funnel plot and Egger regression test if more than 10 studies are included.^[26]^

## 3 Discussion

Although previous studies have reported that ventilator treatment has used for patients with atelectasis, the evidence on its efficacy and safety for atelectasis remains inconclusive. This study will investigate the efficacy and safety of ventilator treatment for patients with atelectasis. It will summarize current evidence on the efficacy and safety of ventilator for atelectasis. Our results of this study will provide helpful evidence for clinical practice and patients. The results from the current systematic review will facilitate evidence-informed decision making and will supply as a clinical guideline towards the present valuable evidence of ventilator for atelectasis for the future researchers, and health policy-makers.

## Author contributions

**Conceptualization:** Zhi-Guo Wang, Jian-Rong Sun, Hai-Wang Sha.

**Data curation:** Zhi-Guo Wang, Jian-Rong Sun, Hai-Wang Sha.

**Formal analysis:** Zhi-Guo Wang.

**Funding acquisition:** Hai-Wang Sha.

**Investigation:** Hai-Wang Sha.

**Methodology:** Zhi-Guo Wang, Jian-Rong Sun.

**Project administration:** Hai-Wang Sha.

**Resources:** Zhi-Guo Wang, Jian-Rong Sun.

**Software:** Zhi-Guo Wang, Jian-Rong Sun.

**Supervision:** Hai-Wang Sha.

**Validation:** Jian-Rong Sun, Hai-Wang Sha.

**Visualization:** Zhi-Guo Wang, Jian-Rong Sun, Hai-Wang Sha.

**Writing – original draft:** Zhi-Guo Wang, Jian-Rong Sun, Hai-Wang Sha.

**Writing – review and editing:** Zhi-Guo Wang, Jian-Rong Sun, Hai-Wang Sha.
